# Roadmap for Effective School-Based Practices to Support Expectant and Parenting Youth: Lessons from the New Heights Program in Washington, DC

**DOI:** 10.1007/s10995-020-02986-4

**Published:** 2020-07-31

**Authors:** Subuhi Asheer, Susan Zief, Ruth Neild

**Affiliations:** Mathematica, P.O. Box 2393, Princeton, NJ 08543 USA

**Keywords:** Case management, Expectant and parenting youth, School-based programs, Advocacy, Program implementation

## Abstract

**Introduction:**

New Heights is a voluntary school-based program that provides a well-defined system of supports for expectant and parenting students in Washington, DC, and was found to be effective at improving educational outcomes. This study explores the program elements and practices that, when used together, improved academic outcomes for New Heights participants and define a possible roadmap for service providers interested in replicating the program’s success.

**Methods:**

The study team collected data through site visits, key informant interviews, staff surveys, program observations, case files, and program materials.

**Results:**

The core design and implementation elements of the New Heights program are (1) placing a trained staff member in the school to provide advocacy, case management, education, and in-kind incentives; (2) bringing community-based service providers into the school; (3) giving trained staff autonomy and a strong grounding in local context; and (4) using a highly collaborative process to hire and support school-based coordinators.

**Discussion:**

Staff and funders interested in improving outcomes for young parents in school could use the experience of New Heights and the key practices that were critical to its success as a guide: (1) ensure that the program is well defined but can be tailored to the needs of schools and students, (2) engage community partners to bring services to participants, (3) hire and train the right staff who are committed to “do whatever it takes,” (4) actively cultivate a culture of collaboration among program staff, and (5) develop buy-in with school staff and illustrate program value.

## Significance

*What is already known*? Programs designed to address the needs of young expectant or parenting mothers often use multiple components and approaches, involving numerous partners. The body of evidence on the effectiveness of some of these programs is growing, but there is still a lack of knowledge about what drives program success.

*What does this article add*? This paper defines and explores the key program design and implementation components of New Heights, an effective school-based program for young parents, which was found to increase credit accumulation; reduce absenteeism; and, to some extent, increase school completion rates. We share key lessons learned for the field based on the program’s successful implementation in Washington, DC.

## Introduction

Programs designed to address the needs of young expectant or parenting mothers often use multiple components and approaches, involving numerous partners (Person et al. 2018). The body of evidence on the effectiveness of some of these programs is growing, but there is still a lack of knowledge about the different components and attributes that coalesce to drive program success (Harding et al. 2020).

Through evaluation efforts supported by the Office of Population Affairs (formerly the Office of Adolescent Health), we identified one Pregnancy Assistance Fund (PAF) funded program—New Heights—that has strong and positive impacts for expectant or parenting young mothers (Asheer et al. 2017b; Zief et al. 2020).

New Heights is a voluntary, school-based program of supports designed to help expectant and parenting mothers and fathers in Washington, DC, Public Schools (DCPS) navigate the challenges of pregnancy, parenthood, and completing high school. Pregnant or parenting students often face judgment in their communities, in class, with their peers, or on their way to and from school. New Heights aims to reduce some of this stigma and stress, increase school engagement and credit accumulation, and build self-sufficiency and resilience. With a 2010 PAF grant, DCPS central office staff refined and expanded New Heights from just two high schools, where it had operated for nearly two decades, to nine of the district’s large, comprehensive high schools that were also in the city’s poorest neighborhoods with the highest rates of teen births (PerryUndem Research/Communication 2013). An evaluation of the program showed that New Heights significantly improved school engagement and credit accumulation among expectant or parenting mothers and, to a lesser degree, improved school completion as well (Asheer et al. 2017b; Zief et al. in press).

This paper discusses the core features and components central to New Heights’ design and implementation that, taken together, could present a roadmap for successfully supporting expectant and parenting mothers and improving their educational outcomes.

## Methods

This study of New Heights implementation examined program delivery in nine schools into which the program expanded in 2010 and incorporated input from staff at the two original New Heights high schools. The research was conducted in accord with prevailing ethical principles and approved by an Institutional Review Board. This manuscript is not based upon clinical study or patient data. The study relied on the following data sources:

1. Site visits, which consisted of key informant interviews with district leaders (n = 2), New Heights program staff (n = 3), school-based coordinators (n = 11), and representatives of several associated community-based providers (n = 12); four focus groups with expecting or parenting young mothers who had participated in the program (n = 29); observations of program delivery in three schools; and review of select participant case files (n = 28). All respondents consented to participate in the study. A team of five site visitors conducted three site visits to Washington, DC, to collect in-depth data on (1) the intended program design for New Heights, (2) the program as implemented at the time of data collection in spring and summer 2015, (3) staff and young mothers’ experiences with the program, and (4) lessons learned from implementation.
2. The New Heights program database, which contains administrative data on program participants and the number and types of workshops delivered through the program.^1^
3. A paper and pencil survey completed by 11 New Heights coordinators (in the nine study schools and the two original New Heights schools).
4. New Heights program materials, including the New Heights manual for program managers (updated January 2015), criteria for vetting and approving community-based providers for educational workshops, video of five-day preservice training of New Heights coordinators, evaluation reports, meeting agendas and notes, and New Heights summit brochures and plans.

Qualitative analysis of interviews, focus groups, observation data, and key program materials described above involved an iterative process of identifying key themes and triangulating data sources. Trained staff used a qualitative analysis software package, Atlas.ti, to facilitate organizing and synthesizing the qualitative data according to themes and key research questions. We used administrative data from the New Heights database to analyze the number of workshops offered and the average workshop attendance rate for female participants in the nine study schools in 2014–2015.^2^ We analyzed the survey data in Excel and calculated descriptive statistics.

In this article, we present the key findings that emerged from our analysis of these data sources.

## Results

**Fig. 1:**
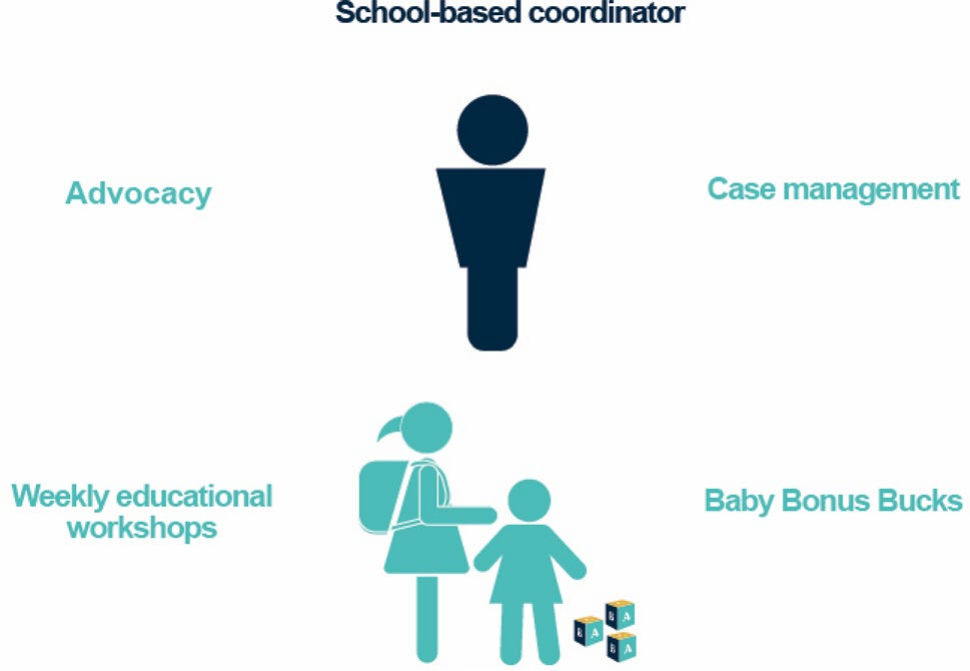
New Heights key components

The New Heights program model is not an off-the-shelf program with a one-size-fits-all curriculum. It clearly defines several core components (Fig. 1) and then relies on a trained staff member, a “coordinator” who is placed in each school, to integrate and customize these in the best way to address the varied needs of young mothers. Based on the data gathered through the study, including our discussions with program leaders, coordinators, expectant or parenting mothers, and community-based providers as well as observations of coordinators, we identified key features that were crucial to the program’s design and successful implementation.

### New Heights Developed a Structured System of Supports that Could be Tailored as Needed

The New Heights program’s key feature is placing a coordinator in every school. The coordinators are trained staff, employed by the district, who operate primarily out of a designated office or classroom space in each school. The coordinator leads the delivery of the program’s multiple components, serving as a resource for his or her school, assessing the academic and personal needs of participating students, and helping them identify concrete strategies to meet their goals.

New Heights does not have a scripted curriculum; rather, coordinators integrate and deliver four main components in their particular schools, which are located in an urban environment rich with invested community partners.

- *Advocacy* Expectant and parenting students typically have needs that make it challenging for them to participate fully in school activities and manage their academic workload. They must make frequent visits to the doctor for which they may have to miss some or all of their school day. Because of their changing bodies, expecting mothers also find their existing uniform shirts or pants no longer fit, or they prefer to wear loose-fitting clothes to avoid undue attention, which can make it difficult to meet the school dress code requirements. Coordinators help reduce stigma and school-based discrimination by educating school staff and students; they also ensure that administrative rules, such as those for excused versus unexcused absences, dress code, or access to an elevator, are modified to accommodate expectant and parenting students.
- *Case management* Coordinators are expected to (1) monitor and promote academic progress for each participant in close collaboration with teachers, counselors, and school administrators; (2) help students manage all logistical or personal challenges—such as referrals to transportation, child care, or housing needs—to ensure that they can attend school every day; and (3) empower students to become self-sufficient by providing them with the tools and skills to advocate for themselves.
- *Educational workshops* Held at least three times a week, lunchtime workshops provide supplemental education on parenting skills, career and financial planning, prenatal care, early childhood development, and healthy relationships. Although the logistics of the workshops are organized by the coordinators, most workshops are delivered by community-based partners. Given the district’s rich environment of nonprofits, New Heights has a well-developed system of community partners to provide students with access to legal assistance, early childhood education, mental health counseling, and college or career planning guidance. Funding permitting, the program also conducts a culminating one-day event, called the Youth Summit, at the end of the year to celebrate participating youth, alumni/ae, and community partners’ achievements.
- *Baby Bonus Bucks* New Heights uses a system of in-kind incentives, or points, called Baby Bonus Bucks to help improve attendance, grades, and class participation. Coordinators develop benchmarks individualized for each student, and students earn these incentives when they meet their goals. Students can use their Baby Bonus Bucks toward “purchasing” items from New Heights staff such as maternity supplies, baby clothes or toys, diapers, formula, and other items.

**Fig. 2:**
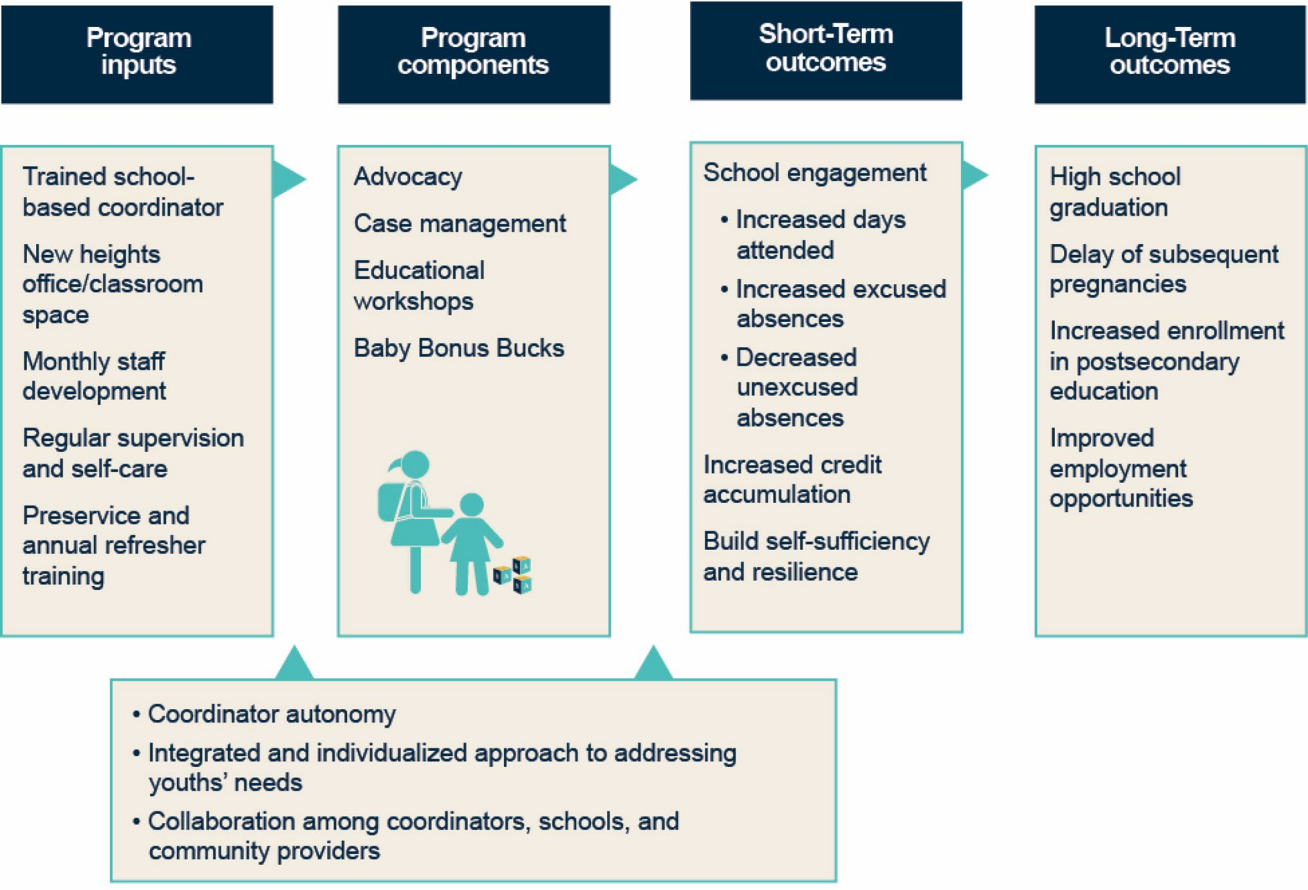
New Heights logic mode

Taken together, these components ultimately aim to help expectant and parenting students achieve educational success (Fig. 2). In the short term, New Heights seeks to increase school engagement through improved attendance and to help students identify their strengths and resources to build self-sufficiency. The program supports students in overcoming the barriers that keep them out of the classroom, thereby working to increase the number of days they attend per year and the number of credits they accumulate. But the program also helps students to understand the criteria for excused absences and the administrative process for having an absence designated as excused, as opposed to unexcused.^3^ These short-term outcomes are expected to lead to long-term improvements, such as increased graduation rates, postsecondary enrollment, employment opportunities, and the delay of subsequent pregnancies. The impact evaluation we conducted found that New Heights had positive impacts on parenting females for all three school engagement outcome domains examined: attendance, credit accumulation, and graduation (Asheer et al. 2017b).

### New Heights’ School-Based Setting Facilitates Meeting Teens Where They Are and Reaching Them Before They Drop Out Due to Pregnancy or Parenting

Identifying and recruiting pregnant and parenting youth are large barriers community-based programs face to improving outcomes in areas with high rates of teen births. Nonprofits and clinics know these youth are there and in need of services, but unless young expectant or parenting mothers or fathers voluntarily walk through nonprofit or clinic doors, it is hard to identify and serve them. New Heights used three key strategies to overcome this challenge.

First, the program is based in the schools, where nurses, counselors, and students can identify pregnant and parenting females and serve them where they are. The impact study found that 75% of the parenting mothers in the New Heights schools take advantage of the program (Asheer et al. 2017b). Such high take-up rates of a school-based offering suggest recruiting and serving young parenting mothers in schools form an effective strategy. As one coordinator emphasized, “Our adolescents, especially those in crisis, need someone (a physical presence) when they need it. Adolescents do not plan out their emergencies, and it is important to be here and to be responsive to them.”

Second, New Heights targeted programming in the schools and areas of the city where teen birth rates are highest. Despite a falling teen birth rate nationwide, teen births continue to be a significant issue for Hispanic and African American females in Washington, DC, with birth rates nearly 25 times that of non-Hispanic white females (Centers for Disease Control and Prevention 2015). These births primarily occur among females living in the neighborhoods with the highest rates of poverty and violence (PerryUndem Research/Communication 2013), making it especially difficult for these young mothers to access the services they need (Rolland 2016; McCoy 2015). The poorest neighborhoods are also home to most of the city’s large, comprehensive high schools. In these large high schools, nearly 10% of females are parenting.^4^ These schools represent a convenient location for expectant and parenting students to receive services in support of their educational attainment.

Finally, New Heights built engagement with community-based providers into its program model. At least three times a week, staff from the region’s rich ecosystem of relevant community-based organizations are invited into the school to conduct lunchtime workshops on a variety of topics (such as contraception, parenting, child development, and so on). Access to these providers during the school day allows students to easily make connections when they need specific resources. In keeping with this approach, New Heights leadership ensured that when making hiring decisions, coordinators had strong connections in the local communities.

### Coordinators Are the Backbone of the Program and Hiring the Right Staff Was Critical to Successful Implementation

For New Heights, the role of the school-based coordinator was central to the program’s operation and highly individualized to the needs of each school and student that the coordinator served. The coordinator’s role required staff who not only supported students but treated them as individuals with strengths and skill sets to help themselves. As one coordinator said, “We don’t provide a service to students—they don’t just come to get the voucher. They come and we teach them how to get a child care voucher. We will step in if there is a problem, but we really want them to learn to advocate for themselves.” For this reason, program leaders balanced two considerations in hiring coordinators: the capacity to deliver an intensive wraparound program and their ability to adapt quickly to the needs of their students and school.

Believing that an interdisciplinary mix of coordinators would build on one another’s strengths and help address different challenges, New Heights leadership staff looked for candidates from varied professional backgrounds whose skills and characteristics would complement each other. Another factor they considered was experience working in DCPS or community-based organizations that would allow coordinators to leverage those prior relationships to more effectively advocate with school administrators on behalf of their students and identify appropriate resources to address challenges. Program leaders also looked for staff who were passionate about helping expectant and parenting students, had experience working with young people (or had been young parents themselves), or had lived and worked in the communities they would be serving and could therefore relate to the challenges expecting or parenting students faced while in school. As one student highlighted, “It is really difficult to try and parent and also do all your schoolwork. There are times when I wanted to give up. You have to care for yourself, and it makes a big difference to know that there are people who care about you.” Finally, when interviewing and meeting candidates, program leaders and veteran staff asked candidates questions to assess personal fit in addition to their professional and academic qualifications. In order to “do whatever it takes” to help their students, a coordinator needed to be adaptable, empathetic, collaborative, an innovative problem-solver, and willing to mentor and coach others (Asheer et al. 2017b).

To match staff with the relevant combination of skills and characteristics for each school, New Heights leadership used a collaborative approach. Although the coordinators were categorized administratively as district central office staff, and the district (not each school) funded their positions, program leaders recognized the importance of involving schools in the hiring process of each coordinator. When expanding to new schools under the PAF grant, New Heights central office staff invited school principals to meet the short-listed candidates and engaged them in the interview process. Based on their discussions, each coordinator candidate and school principal then ranked their top three choices, ensuring that both the schools and the coordinators had buy-in. Two veteran New Heights coordinators were also a critical part of the hiring teams, and their years of experience added important value to the staff recruitment and selection process. New Heights staff also considered any unique student needs or contexts across the expansion schools, emphasizing that in making a hiring decision for each school it was important for them to “see potential candidates through [our] students’ eyes to determine their fit for the program.” The program also offered competitive salaries and relied on a network of existing community partners to attract the right candidate.

### The Coordinators’ Autonomy Is Undergirded by a Strong System of Support from Veteran Staff Who Know the District and the Schools

New Heights central office staff used a highly intentional approach in providing tailored supervision and support to each coordinator on an ongoing basis. First, staff who designed and conducted the 5-day preservice and annual refresher trainings had been with the district for many years, and their deep knowledge of the schools and communities helped coordinators get a clear picture of the ground realities and expectations for their role in each school. Veteran staff walked new coordinators through a typical day, the different types of school staff with whom they would have to build relationships, and the types of information and resources they would need.

In addition, the New Heights program manager served as a supervisor, who was embedded in the schools side-by-side with the coordinators throughout the academic year, learning along with them and serving as an integral and trusted source of support when necessary. Coordinators received ongoing monitoring and one-on-one feedback from the supervisor, who rotated her time among schools year-round. Each week, she aimed to spend four days at a single school to observe its culture, the needs of the students, and the activities of the coordinator. The program manager could then provide more targeted support to each coordinator based on the specific needs and context of their schools and students.

Finally, a collaborative spirit, rather than hierarchy, defined New Heights daily operations. Monthly staff meetings focused on topics coordinators wanted to prioritize, and regularly focused on the self-care strategies coordinators could use to manage the demands of this intensive work.^5^ Outside of the monthly meetings, coordinators interacted frequently for support, guidance, and self-care. The coordinators’ multiple roles—caregiver, mentor, teacher, and counselor—could be overwhelming, causing mental and emotional stress. The monthly meetings and regular communication among the team also provided opportunities to share questions and receive input on specific cases.

## Discussion

The experience of New Heights provides important guiding principles that could be customized by service providers and funders looking to replicate the model and achieve similar outcomes in their own context. The following recommendations draw on the study of the program’s implementation to present lessons learned for helping young mothers overcome significant challenges and thrive in school:

### Ensure that the Program is Well Defined But can be Tailored to the Needs of your Schools and Students

New Heights developed a well-integrated system, led by a school-based coordinator and consisting of advocacy, case management, weekly education, and in-kind incentives, that supports female students in overcoming barriers to attending and completing school. A key aspect of this system is meeting each student where she is and tailoring the system of tools and services to address her needs. These components do not operate in silos—rather, program staff must be given the autonomy and training to customize and combine the different components for the context of their schools and students.

### Engage Community Partners to Bring Much-Needed Services to Participants

Weekly workshops allowed New Heights to provide supplemental education on relevant topics and facilitate in-school connections with community-based providers and services. Developing relationships with invested community partners is important for increasing access and removing barriers to needed services and for providing information on parenting, housing, financial literacy, and reproductive health. Program staff can tailor workshop content and frequency depending on the needs of their students and get their input in selecting the topics and the providers.

### Hire and Train the Right Staff Who are Committed to “Do Whatever it Takes” to Help their Students

The New Heights coordinators formed the backbone of the program. According to participants, New Heights felt like “home,” a safe space for them to shed the stress and stigma of being a young parent. Participants truly appreciated the targeted support and guidance they received from coordinators.

Program leaders should be thoughtful about defining program requirements and expectations for staff early in the process, to help identify and recruit staff with the appropriate skills and personalities to fit the role. Look for staff who are self-motivated, empathetic, and willing to adapt to different school environments and cultures. Envision who the students would want to work with: hiring from local communities, where staff know and can connect with the population, is important. Finally, ensure that the selection process provides opportunities for multiple stakeholders (such as the students, school-based staff, program partners, and others) to share input so that there is buy-in from the start.

### Actively Cultivate a Culture of Collaboration and Ownership Among Program Staff

Fostering a spirit of collaboration and ownership among frontline staff, supervisors, and program leadership can go a long way in building a strong system for implementation and addressing early challenges. For New Heights, this collaborative spirit was driven by highly supportive leaders and experienced peers. Program leaders should incorporate regular opportunities for meaningful engagement as a team, sharing challenges and concerns as a group to brainstorm solutions and encouraging self-care. Clear communication and a safe space to share their perspectives helps staff develop a strong connection to the program model, and to the families they are serving, and believe in what they are trying to achieve together.

### Develop Buy-in with School Staff and Illustrate Program Value

Cultivating and building trust with school staff and local partners were critical for New Heights and formed the foundation for successful program delivery on the ground. Program leaders and staff based in the school should invest in understanding the local context and develop relationships with teachers, administrators, support staff, and students. To secure their support, coordinators can highlight program benefits and find ways in which program staff can relieve burdens for teachers and administrators to help them more easily serve their most challenging students. Volunteering at school events, sharing progress at administrative meetings, and offering assistance and resources can increase buy-in and help meet the needs of young parents.

This study builds on and adds to the existing research with lessons that can be drawn from programs serving expectant and parenting youth.

After its federal grant ended, New Heights had to reduce the number of school-based coordinators and prioritize schools with the greatest number of expectant and parenting students. Staff adjusted their model and sustained their program by using rotating coordinators for schools with a smaller number of potential participants (Asheer et al. 2017a). Service providers and funders looking to replicate the model in smaller schools or less urban contexts may find these adjustments useful when considering modifications to align with their populations’ needs.

This study had some limitations. Its findings are based on the program’s implementation in a specific setting. When considering replication, program leaders should keep in mind that the staffing and structural elements described here were designed for expectant and parenting mothers in schools in Washington, DC, with large student populations and a plethora of community-based resources in an urban setting, so they may not be generalizable to other contexts or populations. In addition, the program aims to serve both mothers and fathers, but the study focused primarily on female participants and thus cannot draw conclusions about the program’s components that would be effective for working with fathers.

## Footnotes

1 Administrative data are not reported in this paper, but findings based on these data were presented in the original report (Asheer et al. 2017b) and in subsequent publications.

2 Although the program serves both expectant and parenting mothers and fathers, the findings in this paper are based on the sample used for the 2017 impact study, which focused on female participants.

3 If a student accumulates 40 unexcused absences in a year, DCPS will disenroll the student. There is no cap on the number of excused absences.

4 This statistic was compiled using data for this study for the nine large public high schools included in the study.

5 “Self-care” refers to strategies, activities, and practices that social workers can engage in on a regular basis to reduce stress, balance work and personal lives, and maintain and enhance short- and longer-term health and well-being (Cox and Steiner 2013).
